# Assessment of Selected Immunophenotypic Markers in Childhood Non‐Hodgkin's Lymphomas in Ghana: A Possible Tool for Effective Stratification of Patients and Assessment of Survival Outcomes

**DOI:** 10.1002/cnr2.70711

**Published:** 2026-10-06

**Authors:** Samuel Asamoah Sakyi, Bernard Gyan, Ernest Adankwah, Ernest Kwasi Adjei, Vivian Paintsil, David Kwabena Adu, Manu Akwasi Owusu, Clement Binwatin Dagungong, Muniru Mohammed Tanko, Kervin Edinam Zogli, Alfred Effah, Samuel Kwame Sopuruchi Agomuo, Samuel Kwarteng, Beatrice Nyantakyiwaa, Edward Osei Gyimah, Harriet Yeboaa Diawuo, George Oduro Owusu, Ebenezer Senu, Oscar Simon Olympio Mensah, Stephen Opoku, Benjamin Amoani, Linda Ahenkorah Fondjo

**Affiliations:** ^1^ Department of Molecular Medicine, School of Medical Sciences Kwame Nkrumah University of Science & Technology Kumasi Ghana; ^2^ Department of Medical Diagnostics College of Health and Well‐Being Kintampo Ghana; ^3^ Department of Medical Laboratory Technology, Faculty of Allied Health Sciences Kwame Nkrumah University of Science & Technology Kumasi Ghana; ^4^ Department of Pathology, Laboratory Services Directorate Komfo Anokye Teaching Hospital Kumasi Ghana; ^5^ Pediatric Oncology Unit, Child Health Directorate Komfo Anokye Teaching Hospital Kumasi Ghana; ^6^ Department of Biomedical Laboratory Sciences, School of Allied Health Sciences University for Development Studies Tamale Ghana; ^7^ Department of Clinical Microbiology, School of Medical Sciences Kwame Nkrumah University of Science & Technology Kumasi Ghana; ^8^ Faculty of Pharmacy and Pharmaceutical Sciences Kwame Nkrumah University of Science & Technology Kumasi Ghana; ^9^ Department of Biological Sciences, School of Natural Sciences and Mathematics The University of Texas at Dallas Richardson Texas USA; ^10^ Division of Clinical Immunology and Rheumatology University of Alabama at Birmingham Birmingham USA; ^11^ Department of Biomedical Science, School of Allied Health Sciences University of Cape Coast Cape Coast Central Region Ghana

**Keywords:** BCL‐2, CD25, CD3, immunohistochemistry, immunophenotyping, non‐Hodgkin lymphoma, survival

## Abstract

**Background:**

Non‐Hodgkin lymphomas (NHL) represent a heterogeneous group of malignancies with varying molecular and immunophenotypic characteristics. In Ghana, NHL is the third most common childhood cancer, highlighting the need for precise diagnostic approaches to improve survival rates. The identification of immunophenotypic biomarkers expressed by both healthy and neoplastic cells of the immune systems has been shown to provide information on the diagnostic and prognostic characterization of tumors leading to personalized treatment of NHL. We evaluated the immunophenotypic profiles of pediatric NHL and their correlation with survival outcomes.

**Aims:**

This study aimed to evaluate the immunophenotypic characteristics of three subtypes of non‐Hodgkin lymphoma (NHL) and their associated survival outcomes among children diagnosed at Komfo Anokye Teaching Hospital, Kumasi.

**Methods:**

In this retrospective study, 38 NHL patients were recruited from the Komfo Anokye Teaching Hospital from 2018 to 2022. Clinico‐pathological characteristics and treatment outcome were obtained in patients' folders. Formalin fixed paraffin embedded tissues were histologically examined and immunohistochemistry performed using monoclonal antibodies targeting *CD3*, *CD25* and *BCL‐2*. The Kaplan–Meier method was used to obtain survival curves and compared by the log‐rank test. Statistical analysis was performed using SPSS (version 26.0) and XLSTAT.

**Results:**

Females accounted for 55.3% of cases, with most patients being under 10 years. Within the first‐year post‐diagnosis, 30.6% of children died, and 27.8% abandoned treatment. *BCL‐2* was the most frequently expressed marker (55.3%), with co‐expression observed across all lymphoma types and age groups. Survival analysis was performed on 21/38 participants with complete follow‐up data. The median survival time was 22 months (95% CI: 3.96–40.11), with survival rates declining from 76% at 10 months to 20% at 40 months. Factors significantly associated with poor survival included *BCL‐2* expression (poorer survival) and extranodal spread (*p* < 0.05).

**Conclusion:**

The survival rate for NHL patients in Ghana is very low. *BCL‐2* was the most expressed marker, significantly influencing survival outcomes. The extent of tumor spread also influenced survival outcomes. Identifying prognostic factors for early diagnosis and treatment is essential to improving survival outcomes for NHL patients in this setting.

## 1 Introduction

Non‐Hodgkin Lymphoma (NHL) remains a significant public health concern worldwide. According to the 2022 Global Cancer Observatory (GLOBOCAN) report, NHL was ranked as the 10th most common neoplasm and the 11th leading cause of all cancer deaths globally, occurring in males more often than females [1]. Approximately 84% of NHL cases in children below 16 years are reported in Latin America, Africa, Asia, the Caribbean, and Polynesia [2, 3]. Moreover, it is estimated that about 80% of children who develop these neoplasms live in developing countries [4]. In Ghana, NHL is ranked the 3rd leading cause of childhood cancer deaths [5] and accounts for an estimated 7% of childhood neoplasms [3, 6]. A 3‐year review report on childhood malignancies at Korle Bu Teaching Hospital in Ghana showed that lymphomas accounted for over 60% of cases, with the most common type being Burkitt lymphoma.

NHLs are described as heterogeneous and their close morphological resemblance adds a degree of complexity to accurate characterization and differentiation [7]. The WHO has classified over 50 sub‐types of NHL taking into account the clinical features of presentation, morphological characteristics of tumor cells, expression of distinct antigenic markers, and genetic features present in tumor cells. Extensive evaluation that incorporates these tumor characteristics is required for definitive diagnosis and classification which are crucial for accessing effective and curative therapy [8, 9, 10].

The identification of immunophenotypic biomarkers expressed by both healthy and neoplastic cells of the immune systems has been shown to provide information not only on the diagnostic characterization of tumors but also applied in both prognostic and personalized treatment of NHL [11, 12]. The expression of cluster of differentiation 3 (*CD3*), 25 (*CD25*), and B cell lymphoma‐2 (*BCL‐2*) biomarkers has been explored through immunophenotypic techniques. These biomarkers have wide applications in establishing tumor cell lineage (*CD3*), assessing host response in the tumor microenvironment (*CD25*), and evaluating resistance of tumor cells to apoptosis and chemoresistance (*BCL‐2*).

Advances in immunophenotypic biomarker discovery and targeted therapy have led to improved clinical outcomes of patients with lymphoid tumors [13, 14]. In developed countries where immunophenotypic testing has been incorporated in routine pathological services, there is enough evidence of improved clinical outcomes and overall survival (OS) of patients with NHL. However, this is not the case in the management of NHL patients in resource‐limited countries like Ghana. The combination of clinical and available histological information is usually used as a means of managing NHL patients at the first point of call [7]. Moreover, procedures initiated by physicians for the treatment of childhood NHL in most African countries, including Ghana, usually proceed without additional diagnostic procedures such as cytogenetic testing or molecular assays being requested, due to the lack of well‐resourced laboratories, high costs, and limited trained personnel [15, 16]. The WHO emphasizes that morphological assessment alone is insufficient for accurately diagnosing and classifying NHL subtypes, as it risks discordant reports even among pathologists, complicating the identification of rare and heterogeneous cases and hindering the planning of targeted therapy. Thus, many children with NHL might be treated without a definitive pathological diagnosis and may either be undertreated or given unnecessarily higher doses that are toxic and harmful to their health.

In Ghana, the lack of cancer registries and dedicated pediatric oncology centers leads to underreporting of childhood cancer cases, including NHL. Diagnostic and prognostic biomarkers are not routinely utilized. Moreover, the association between immunophenotypic profiles and survival outcomes among children with NHL remains understudied in Ghana. Against this background, the current study evaluated the immunophenotypic characteristics of three NHL subtypes and survival outcomes among children at the Komfo Anokye Teaching Hospital.

## 2 Materials and Methods

### 2.1 Study Design and Setting

This retrospective cohort study was conducted among NHL patients at the clinical oncology department of the Komfo Anokye Teaching Hospital (KATH). KATH is the second‐largest hospital in Ghana and serves as a referral and teaching facility situated in Kumasi, the capital of the Ashanti region.

### 2.2 Samples and Data Collection

Formalin‐fixed, paraffin‐embedded (FFPE) tissue blocks were retrieved from the pathology laboratory archives of KATH. The selected tissue blocks belonged to children under 18 years of age who were clinically diagnosed with NHL between 2018 and 2022. Demographic data, clinical summaries, tissue source and type, as well as histological findings, primarily based on hematoxylin and eosin (H&E) staining were recorded. A total of 133 cases were identified and included for downstream analysis. Follow‐up was conducted at the pediatric oncology unit to collect additional clinicopathological and treatment‐related information for the respective cases.

### 2.3 Inclusion and Exclusion Criteria

Children under 18 years of age who were clinically diagnosed and histologically confirmed with non‐Hodgkin lymphoma (NHL), and who had received continuous chemotherapy for at least 1 month, were considered eligible for inclusion. Cases were excluded if data were incomplete, tissue samples were inadequate for analysis, or if there was a history of HIV co‐infection (Figure 1).

**FIGURE 1:**
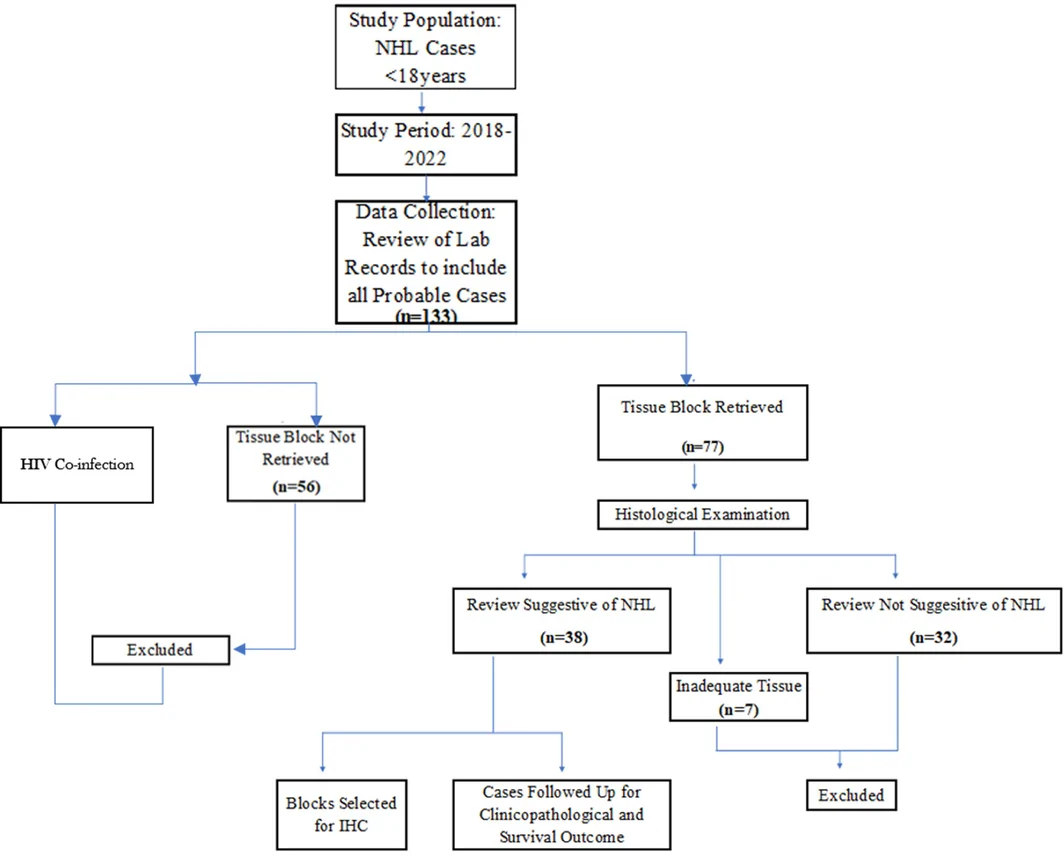
Procedure for case identification and selection for study.

### 2.4 Histological Examination

Using a rotary microtome, 4 μm thin sections were cut from each block and fixed on a glass slide. The slides were kept in an oven at a temperature of 60°C for 60 min to ensure complete drying and to remove excess wax from the section. Commercially prepared Mayer's Hematoxylin and Eosin (H&E) (Sigma‐Aldrich, USA) was used for progressive staining of the slides as per the KATH standard operating procedures (SOP). An automated staining device, Leica ST5010 Autostainer XL (Leica Biosystems, Germany) was used by running through a complete cycle of deparaffinization in three (3) changes of xylene, hydration in decreasing grades of alcohol (ethanol) until the tissue was returned to water and progressive staining with H&E. The tissues were dehydrated and mounted in DPX.

### 2.5 Slide Review and Reporting

Two (2) independent pathologists at the Department of Pathology, at the Kwame Nkrumah University of Science and Technology reviewed and reported the status of each slide. Each pathologist reported independently and was blinded to previous histology reports. Any discordant report was reviewed by a third pathologist to determine the final status of the slide. The status of each slide was assigned as negative or positive to indicate the absence or presence of histomorphological features consistent with non‐Hodgkin lymphoma, including diffuse effacement of normal nodal architecture, atypical lymphoid proliferation, increased mitotic activity, and loss of normal germinal center structures (Figure 2). Thirty‐eight (38) cases were adjudged positive and suitable for immunohistochemical analysis.

**FIGURE 2:**
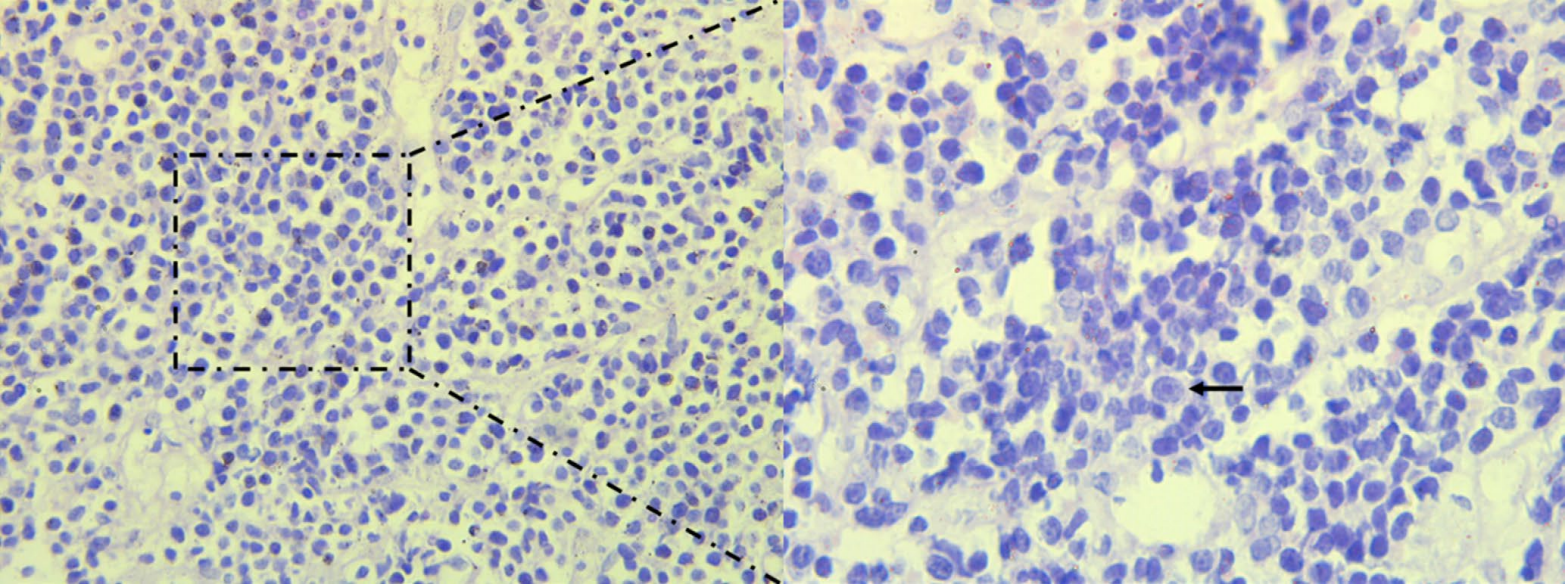
H&E staining demonstrating the presence of NHL cells in formalin‐fixed, paraffin‐embedded tissue sections. NHL cells were observed under ×40 objective and NHL cells were observed under ×100 objective, showing diffused neoplastic cells with dense chromatin and a large nuclei to cytoplasmic ratio.

### 2.6 Clinical Aggressiveness

Low‐grade NHL comprised indolent lymphomas, whereas high‐grade NHL comprised aggressive lymphomas as classified in the clinical records. Burkitt lymphoma was analyzed as a separate category because of its distinct clinicopathologic features and highly aggressive clinical behavior. These categories were used for comparative analyses and do not represent formal WHO or ICC diagnostic classifications.

### 2.7 Immunohistochemistry Examination (IHC)

Serial sections (4 μm) from the 38 cases adjudged positive and suitable for IHC were made onto positively charged slides. Tissues were deparaffinized by clearing through two (2) changes of xylene and hydrated through decreasing grades of alcohol and water. The heat induced epitope retrieval (HIER) method was used to recover antigens. Using a microwave, antigens were retrieved in 10 mM Trisodium Citrate Buffer (10 mM Trisodium Citrate, 0.05% Tween, pH 6.0), heated at 95°C for 40 min and washed in Phosphate Buffered Saline (PBS, pH 7.2). Slides were blocked from endogenous peroxidase activity by incubating in a solution of 3% H_2_O_2_ for 5 min and washed in PBS. 1% Bovine Serum Albumin (BSA) diluted in PBST was used to block non‐specific protein binding at 37°C for 1 h and washed in PBS.

Immunostaining was performed using mouse monoclonal antibodies obtained from Santa Cruz Biotechnology Inc., specific for *CD3* (PC3/188A: sc‐20 047), *CD25* (IL‐2Ra (IL2R.1): sc‐57 297), and *BCL‐2* (*BCL‐2* (100): sc‐509). Primary antibody staining was detected using an HRP‐conjugated secondary antibody kit. Reaction signal was detected using a diaminobenzidine (DAB) chromogenic substrate kit. Mayer's Hematoxylin was applied for counterstaining and mounted in DPX for evaluation. All antibody targets demonstrated satisfactory signals in known negative and positive controls.

Known positive control tissues (tonsil for *CD3*, reactive lymph node for *BCL‐2* and *CD25*) were included in each staining batch, while negative controls were obtained by omitting the primary antibody. Interpretation followed a semi‐quantitative scoring system based on the intensity and proportion of stained tumor cells.

### 2.8 IHC Evaluation and Semi‐Quantitative Reporting

Expression signals were evaluated and reported under a light microscope. Two (2) independent pathologists examined and scored both the intensity of signal developed and proportion of tumor cells stained in each case. The entire section was assessed and evaluated at ×100 power field before assigning the final score. Mean score from the two (2) pathologists was determined in each case. Discrepant reports were re‐evaluated by a third pathologist and a final report assigned upon agreement between them. Signal intensity was scored as follows: 0 (no staining observed), +1 (mild staining), +2 (moderate staining), and +3 (strong staining). The proportion of tumor cells stained was scored as follows: 0 (no staining), +1 (< 20% of tumor cells show staining), +2 (20%–40% of tumor cells show staining), +3 (41%–60% of tumor cells show staining), +4 (61%–80% of tumor cells show staining), +5 (> 80% of tumor cells shows staining). Final IHC positivity score was determined by the sum of the scores obtained for both signal intensity and proportion of tumor cells expressing the signal. IHC scores were ranked as follows: no expression (0), low expression (1–2), moderate expression (3–5), and high expression (≥ 6) (Figure 3).

**FIGURE 3:**
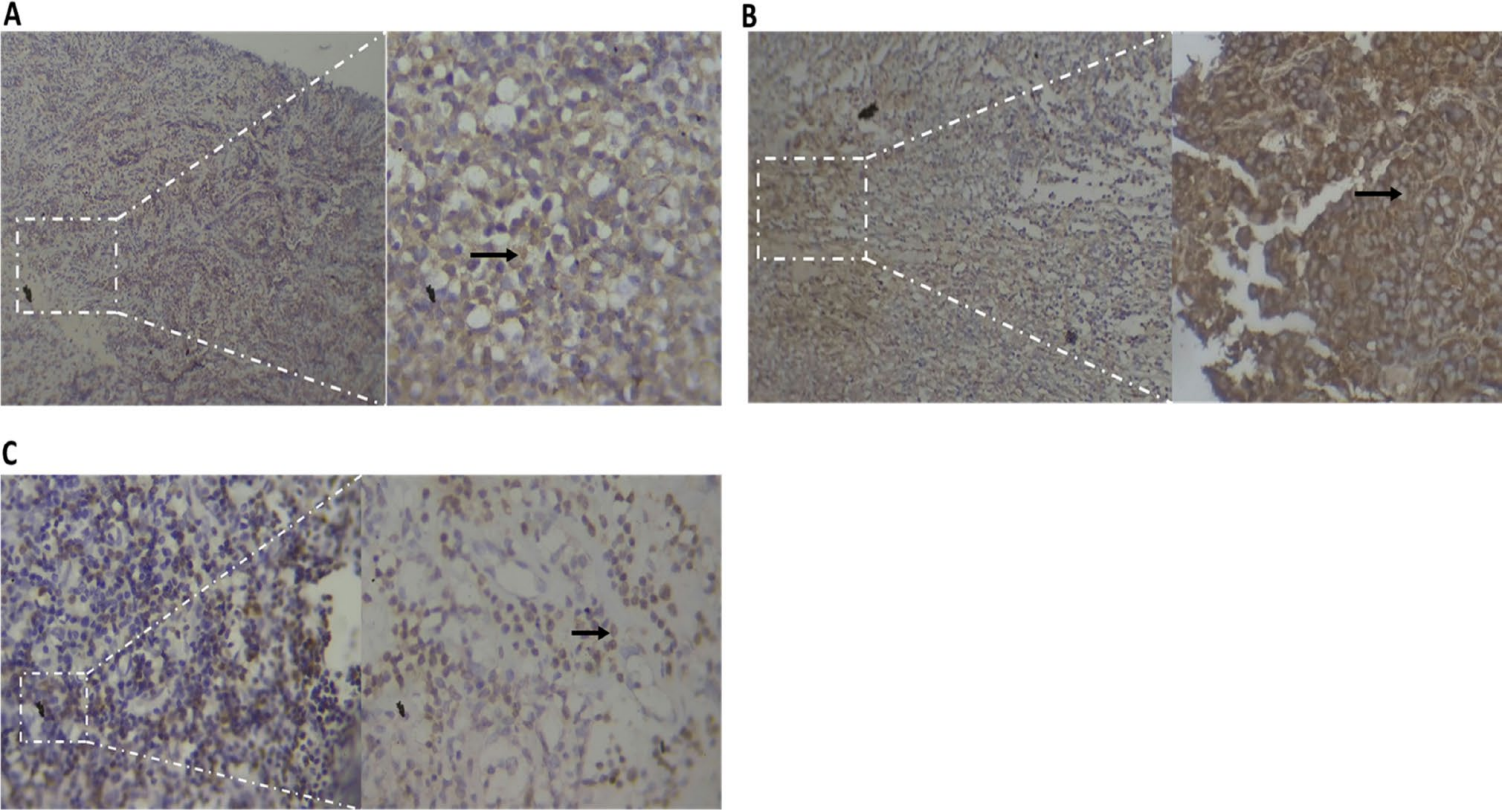
IHC expression of CD3, BCL‐2, and CD25 in NHL tissue samples. Representative micrographs of (A) CD3, (B) BCL‐2, and (C) CD25 expression at magnifications ×40 and ×100 as indicated. Positive immunostaining is visualized as brown diaminobenzidine (DAB) chromogen. Black arrows indicate representative cells or areas demonstrating positive staining against the blue/purple hematoxylin counterstain.

### 2.9 Definition of Term

Palliative care was defined as comfort‐focused supportive care including symptom management, pain relief, and psychosocial support, provided primarily to the patients with advanced conditions or those unable to tolerate curative treatment.

### 2.10 Statistical Analysis

Data obtained were analyzed using statistical package for social sciences (SPSS, version 26.0) and XLSTAT. Frequency of expression of immunophenotypic biomarkers and histological subtypes of NHL lymphoma was determined using frequency distributions and percentages. The Kaplan–Meier test was used to determine the overall survival (OS) among participants, which was calculated from the date of diagnosis until death or last documented follow up. The log‐rank test was used to determine clinicopathological and immunophenotypic factors associated with overall survival of participants. In all cases, a 95% CI was used and a *p* < 0.05 was considered statistically significant.

## 3 Results

The study involved 38 participants, 36.8% of which were below 7 years old, 36.8% were between 7 and 10 years, and 26.3% were over 10 years. There were more females (55.3%) than males (44.7%). 2.7% of participants were underweight, 8.1% were overweight, and 2.7% were obese at the time of diagnosis (Table 1).

**TABLE 1:** Sociodemographic characteristics of NHL pediatric patients (*n* = 38).

Variable	Frequency (*n* = 38)	Percentage (%)
Age group (years)		
< 7	14	36.8
7–10	14	36.8
> 10	10	26.3
Gender		
Female	21	55.3
Male	17	44.7
BMI category		
Underweight	1	2.7
Normal	32	86.5
Overweight	3	8.1
Obese	1	2.7

*Note:* Data presented frequency and percentage (%).

Abbreviation: BMI, body mass index.

### 3.1 Clinical Characteristics of Study Participants

The duration of symptoms before diagnosis was almost evenly distributed, with 51.4% of patients presenting within 1–10 weeks and 48.6% presenting after more than 10 weeks. Most of the tumors were located in the cervicocephalic region (63.2%) with advanced stages (Stages III and IV) of presentation: 13.2% and 18.4% respectively. 57.9% of cases were not graded with no attributed reasons. The majority of cases (76.3%) had both nodal and extra nodal involvement with only a few tumors localized in lymph nodes (5.3%). Only a few participants received radiotherapy (2.6%) and palliative care (39.5%). In the course of chemotherapy and patient management, 27.8% abandoned treatment, 28% died on admission, and 2.8% died shortly after discharge (Table 2).

**TABLE 2:** Clinical characteristics and treatment outcomes of NHL pediatric patients.

Variable	Frequency (*n* = 38)	Percentage (%)
Comorbidity		
No	37	97.4
Yes	1	2.6
Duration of symptom (weeks)	**13.3 ± 10.8**	
1 to 10	18	51.4
Above 10	17	48.6
Site		
Cervicocephalic region	24	63.2
Abdominopelvic region	12	31.6
Thoracic region	2	5.3
Stage		
I	1	2.6
II	3	7.9
III	5	13.2
IV	7	18.4
Not graded	22	57.9
Nodal/Extra nodal involvement		
Extra nodal	7	18.4
Nodal	2	5.3
Both nodal and extra nodal	29	76.3
Radiotherapy		
No	37	97.4
Yes	1	2.6
Palliative care		
No	23	60.5
Yes	15	39.5
Relapse		
No	37	97.4
Yes	1	2.6
Clinical outcome		
Abandoned	10	27.8
Survived and discharged	14	38.8
Died on admission	11	30.6
Died on discharge	1	2.8

*Note:* Data presented as frequency and percentages (%). Bold values represent the mean and standard deviation of the duration of symptoms in weeks (mean = 13.3, SD = plus or minus 10.8).

### 3.2 Biomarker Expression in Various Clinical Aggressiveness of Lymphoma

From Table 3, *BCL‐2* was the most expressed (55.3%) biomarker among cases. *CD3* was expressed in 36.8% of cases, whilst *CD25* was least expressed (5.3%). Co‐expression was reported at varying degrees among cases, with 5.3% cases expressing all makers simultaneously.

**TABLE 3:** Biomarker (*CD3*, *BCL‐2*, and *CD25*) expression among NHL patients.

Biomarker expression	Frequency (*n* = 38)	Percentage (%)
*BCL‐2*	21	55.3
*CD3*	14	36.8
*CD25*	2	5.3
*BCL‐2*, *CD3*	11	28.9
*BCL‐2*, *CD25*	2	5.3
*CD3*, *CD25*	2	5.3
*BCL‐2*, *CD3*, *CD25*	2	5.3

*Note:* Data presented as frequency and percentages (%); Markers were not mutually exclusive; co‐expression occurred.

Figure 4 shows the overall IHC score indicating biomarker expression levels (low, moderate, high) among cases. For *BCL‐2*, 47.6% of the cases exhibited moderate expression, while 52.4% showed high expression. *CD3* showed low expression in 57.1% of the cases and moderate expression in 35.7%, with high expression in 7.1%. *CD25* was expressed only at moderate levels.

**FIGURE 4:**
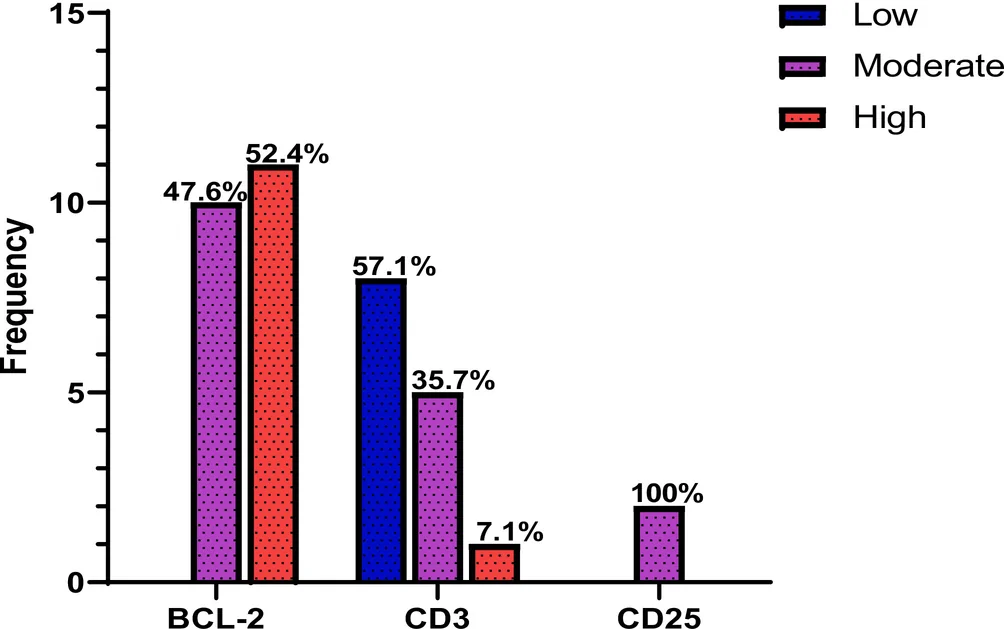
Distribution of immunohistochemical expression levels for *CD3*, *BCL‐2*, and *CD25* biomarkers in NHL Cases (*n* = 38). Bar charts show the proportion of cases exhibiting low, moderate, and high expression for each biomarker.

### 3.3 Biomarker Expression Stratified by Clinical Aggressiveness

Biomarker expression was stratified by clinical aggressiveness of lymphoma, including low‐grade, high‐grade, and Burkitt lymphomas. As shown in Table 4, *BCL‐2* showed high expression in low‐grade cases (76.9%), moderate expression (50.5%) in high‐grade cases, and low expression (36.4%) in Burkitt lymphomas. *CD3* was less frequently expressed in low‐grade NHL (38.5%) and high‐grade NHL (50.0%), with Burkitt lymphoma showing the lowest expression at 18.2%. *CD25* was rarely expressed across all subtypes, with 15.4% in low‐grade cases.

**TABLE 4:** Antigen expression stratified by clinical aggressiveness of NHL.

Biomarker	Low grade (*n* = 13)	High grade (*n* = 14)	Burkitt lymphoma (*n* = 11)	*p*
*BCL‐2*				0.122
No	3 (23.1)	7 (50.0)	7 (63.6)	
Yes	10 (76.9)	7 (50.0)	4 (36.4)	
*CD3*				0.259
No	8 (61.5)	7 (50.0)	9 (81.8)	
Yes	5 (38.5)	7 (50.0)	2 (18.2)	
*CD25*				0.131
No	11 (84.6)	14 (100.0)	11 (100.0)	
Yes	2 (15.4)	0 (0.0)	0 (0.0)	

*Note:* Data presented frequency (%). Markers were not mutually exclusive; individual cases could express more than one marker. *p*‐Value generated from chi‐square or fisher's exact test (*n* < 5).

### 3.4 Survival Among NHL Patients

Of the 38 participants included in the overall cohort, survival analysis was restricted to 21 participants with complete follow‐up data. The Kaplan–Meier test was run to determine the overall survival (OS) time among participants. As depicted in Figure 5, the overall median survival time was 22 months [95% CI (3.96–40.11)] from the date of diagnosis. The survival rates at 10th, 20th, 30th and 40th months were 76% [95% CI (58.4–100.0)], 61% [95% CI (40.0–92.7)], 40% [95% CI (16.4–100.0)], 20% [95% CI (3.8–100.0)].

**FIGURE 5:**
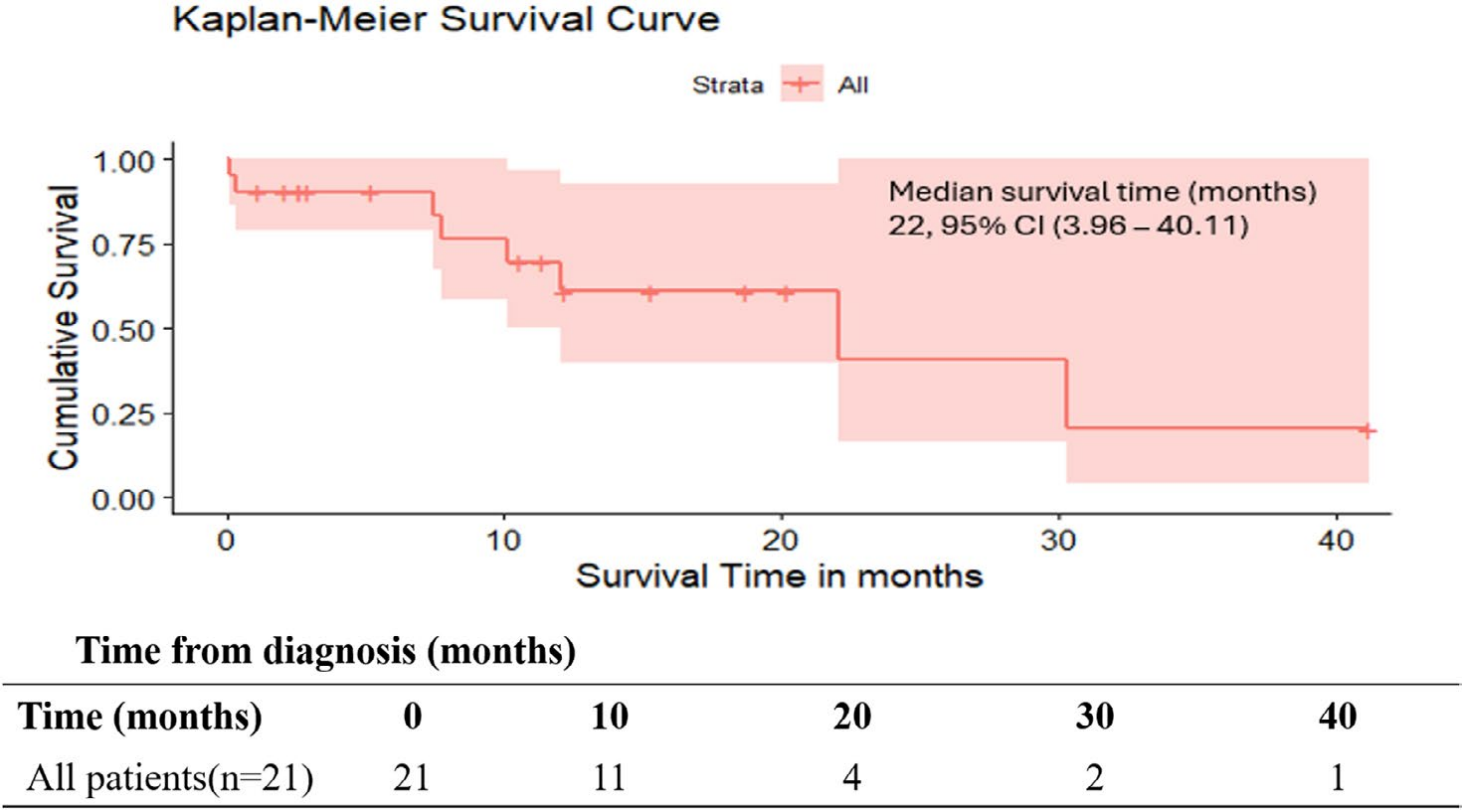
Kaplan–Meier Overall Survival Function Curve with number‐at‐risk tables in NHL Cancer Patients. Wide confidence intervals at later time points reflect the small number of patients remaining at risk, resulting in greater uncertainty around the survival estimates.

The survival rate was higher among patients with high‐grade lymphoma 66% [95% CI (35.2–100.0)] compared to Burkitt lymphoma 38% [95% CI (8.4–100)] and low‐grade lymphomas (0%). However, there was no statistically significant difference in survival rates between the various histological subtypes (*p* = 0.620) (Figure 6).

**FIGURE 6:**
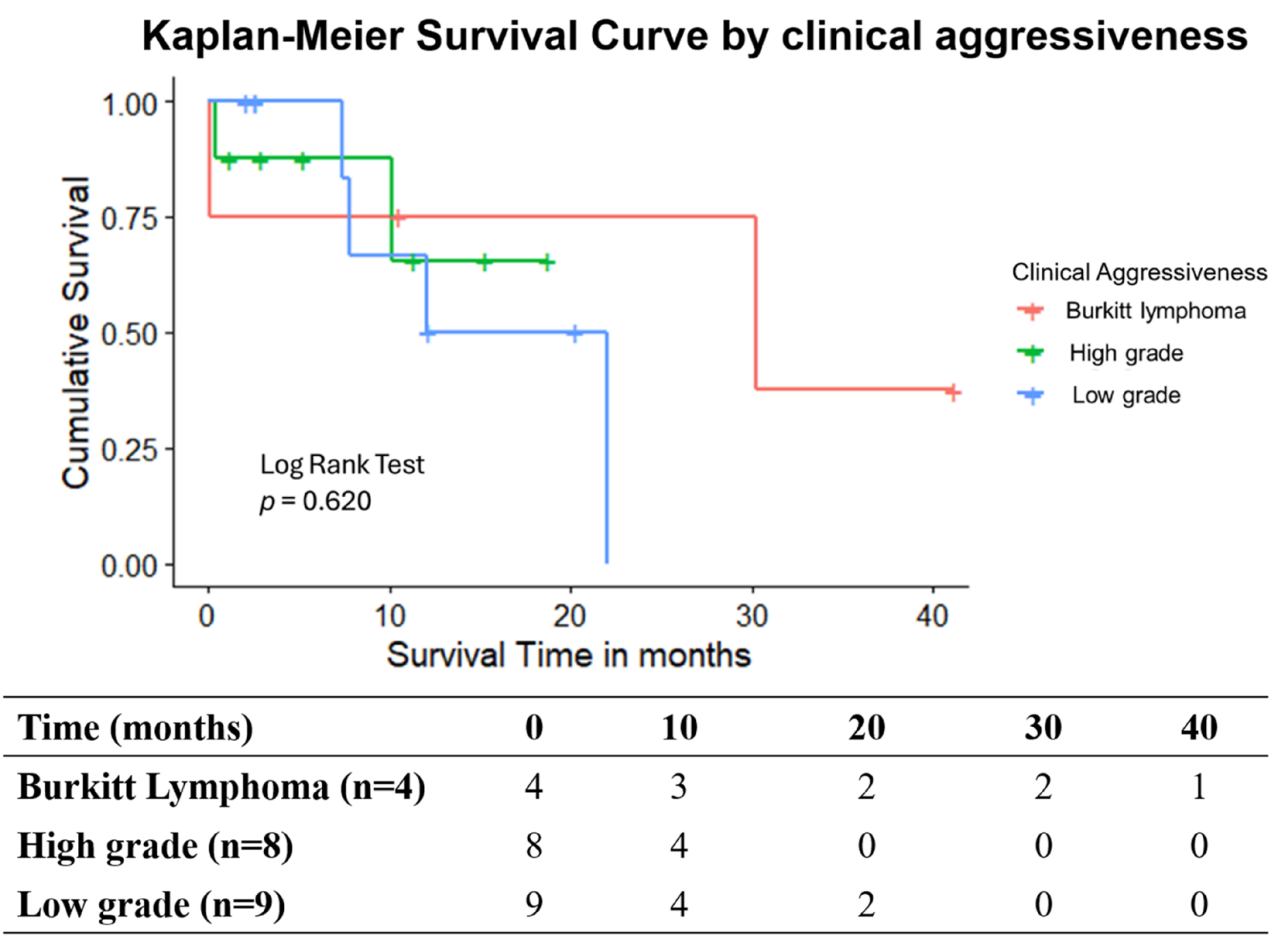
Cumulative Survival of NHL Based on clinical aggressiveness. Tick marks (+) indicate censored observations (patients alive or lost to follow‐up). Wide confidence intervals at later time points reflect the small number of patients remaining at risk, resulting in greater uncertainty around the survival estimates.

### 3.5 Factors Associated With Survival Among Lymphoma Patients

Using the Log Rank test, the association between survival and sociodemographic and clinical characteristics of participants was assessed. As shown in Table 5, there was no significant association between age, gender, duration of symptoms, *CD3* expression, *CD25* expression, clinical aggressiveness of lymphoma, and survival (*p* > 0.05). However, *BCL‐2* expression (*p* = 0.030) and nodal/extra nodal involvement (*p* < 0.001) were significantly associated with survival.

**TABLE 5:** Association of sociodemographic and clinical factors with survival using log rank test.

Variable	Frequency (*n*, %)	Median OS (95%, CI) (months)	*p*
Age group (years)			0.187
< 7	7 (33.3)	12 (7.98–16.09)	
7–10	9 (42.9)	—	
> 10	5 (23.8)	—	
Gender			0.972
Female	12 (57.1)	22 (7.39–36.69)	
Male	9 (42.9)	—	
Duration of symptom (weeks)			0.089
1–10	13 (61.9)	12 (4.23–19.85)	
Above 10	8 (38.1)	30 (1.01–59.57)	
*BCL‐2* Expression			**0.030**
No	9 (42.9)	30 (17.08–43.49)	
Yes	12 (57.1)	12 (6.23–17.84)	
*CD3* Expression			0.149
No	13 (61.9)	30 (3.40–57.18)	
Yes	8 (38.1)	—	
*CD25* Expression			0.257
No	19 (90.5)	22 (4.18–39.88)	
Yes	2 (9.5)	—	
Clinical Aggressiveness			0.622
NHL	9 (42.9)	12 (3.48–20.59)	
High Grade	8 (38.1)	—	
Burkitt lymphoma	4 (19.0)	30 (0.0–75.52)	
Nodal/extra nodal involvement			**< 0.001**
Extra nodal	5 (23.8)	—	
Nodal	1 (4.8)	—	
Both nodal and extra nodal	15 (71.4)	26 (4.59–39.48)	

*Note:* Analysis includes 21 participants with complete follow‐up data (diagnosis date, outcome date, and vital status). Seventeen participants were excluded due to incomplete date information or loss to follow‐up. Median OS values are reported as ‘—’ for groups with very small dead frequencies. Log Rank test *p*‐values were presented; *p* < 0.05 was considered statistically significant. Bolded *p*‐values were statistically significant.

Abbreviations: NHL, non‐Hodgkin lymphoma; OS, overall survival.

There was no significant association between biomarker expression levels, clinical outcome of patients, and site of tumor as shown in Table 6.

**TABLE 6:** Association between antigen expression, clinical outcome and tumor specimen site.

Antigen expression	Site of specimen			*p*
	Cervicocephalic (*n* = 24)	Abdominopelvic (*n* = 12)	Thoracic (*n* = 2)	
*BCL‐2*				0.883
No	10 (58.8)	6 (35.3)	1 (5.9)	
Yes	14 (66.7)	6 (28.6)	1 (4.8)	
*CD3*				0.524
No	15 (62.5)	7 (29.2)	2 (8.3)	
Yes	9 (64.3)	5 (35.7)	0 (0.0)	
*CD25*				0.820
No	23 (63.9)	11 (30.6)	2 (5.6)	
Yes	1 (50.0)	1 (50.0)	0 (0.0)	
Clinical outcome				0.904
Abandoned treatment	5 (50.0)	4 (40.0)	1 (10.0)	
Survived and discharged	9 (64.3)	4 (28.6)	1 (7.1)	
Died on admission	7 (63.6)	4 (36.4)	0 (0.0)	
Died on discharge	1 (100.0)	0 (0.0)	0 (0.0)	

*Note:* Data presented as frequency (%). *p <* 0.05 is considered statistically significant.

## 4 Discussion

In Ghana, NHL has been reported to be a common ailment among children [17]. This study evaluated the immunophenotypic expression of *CD3*, *CD25*, and *BCL‐2* biomarkers and survival outcomes in Ghanaian children histologically diagnosed of NHL.

In this study, 73.6% of participants were 10 years or younger, while 26.3% were over 10 years.

This finding is similar to a study by Pedrosa et al. in Northeast Brazil where the incidence of NHL rises uniformly with age affecting younger children and not commonly diagnosed among children below 2 years [18, 19, 20]. Younger age distribution are risk factors associated with immune disorders such as NHL and this could explain for the higher incidence of NHL in the early life of individuals [20]. The current study observed a 1.2:1 female to male ratio. This finding is contrary to study reports by Adomako et al., Choeyprasert et al., Eke et al., and Hesseling et al. in Ghana, Thailand, Nigeria and Cameroon [20, 21, 22, 23], where there was male predominance. There is considerable evidence indicating the role of estrogen on the proliferation of lymphoma cell whiles downregulating immune response [24]. This assertion could not be the case in our study because estrogen is predominantly associated among females in the reproductive age. Most of the study participants were below 10 years and their estrogen levels may not be considered as significant risk factors for NHL. However, Adinma et al. reported similar findings in the northern region at the Tamale Teaching Hospital (TTH) in Ghana where females dominated over their male counterpart [25]. The TTH and KATH where these studies were conducted, share similar geographical characteristics and draws patients from shared communities in the middle and northern belt of the country. Contrary to the findings by Adomako et al. [20] at the Korle‐Bu Teaching Hospital (KBTH) which is in the southern part of Ghana, the differences in these findings could explain the variations in demographic and clinical characteristics associated with NHL in children.

The clinical outcome of patients after initiation of chemotherapy was classified as abandoned treatment, survived and discharged, died on admission, and died after discharge. We observed that 11 (30.6%) patients died while on admission for chemotherapy, and 10 (27.8%) abandoned treatment. Similar to our findings, a 22% death rate was recorded among Kenyan children undergoing NHL chemotherapy, while 16% relapsed and died after discharge [26]. Hesseling et al. reported a much lower death rate (9.5%) among participants on admission and treatment abandonment (4.5%) [23]. Poor healthcare delivery systems associated with developing countries, combined with challenges in timely access to care, likely contribute to the high mortality rates recorded in these studies. Notably, in our data, patients presenting after more than 10 weeks had a longer median survival (30 months) compared to those presenting within 1–10 weeks (12 months). This suggests that the duration of symptoms alone does not explain mortality, and that other factors such as tumor biology, treatment access, and healthcare quality may play a more decisive role. The delay in reporting to hospitals for care and poor healthcare delivery systems associated with developing countries could account for the high mortality rates recorded in these studies. The high rate of abandoning treatment highlights the gaps associated with managing childhood cancers in developing countries. Poverty and the social burden associated with chemotherapy are common reasons why patients abandon treatment.

We observed that the distribution of biomarker expression varied significantly among children. The majority (55.3%) of patients expressed *BCL‐2*, 36.8% expressed *CD3* while 5.3% expressed *CD25*. The biomarkers were also co‐expressed in varying proportions. The pattern of expression of biomarkers corroborates with the well accepted heterogeneous nature of NHL tumors [27], with a varied degree of apoptosis (*BCL‐2* expression), cell lineage involvement (*CD3* expression), and immune regulatory responses (*CD25* expression). We found out that 14 (36.8%) of the lymphomas demonstrated *CD3* immunoreactivity. Given that *CD3* broadly marks T lymphocytes and cannot, in isolation, distinguish neoplastic T cells from reactive or background T‐cell infiltrates, CD3 positivity alone was not regarded as definitive evidence of T‐cell lineage without clear localization of staining to the neoplastic cell population. Therefore, these findings should be interpreted cautiously. The remaining cases did not express this marker, but because no B‐cell–specific markers were included in the study, their lineage could not be definitively determined. Given the epidemiological predominance of B‐cell lymphomas such as Burkitt lymphoma in African children, it is plausible that many of these unstained tumors were of B‐cell origin; however, this remains unproven within the scope of our study. The proportion of T‐cell lymphomas in this study (36.8%) is higher than the 10.6% previously reported in Ghana [20], and 21.7% in Dhaka [28], but lower than the *CD3* expression of 43.2% of all lymphoma cases observed in Iran [29]. There is a significant difference in the rates reported in our study and that of Adomako et al. even though they were both of Ghanaian origin [20]. This difference is likely due to methodological differences, particularly the study's focus on children, sample size variation, and case composition. While geographical and ethnic factors may contribute to differing lymphoma patterns through environmental exposures, infectious cofactors, or host genetics, these are more likely to influence disease distribution and presentation. The differences may also indicate the significant variation in childhood NHL lymphoma from adult ones as reported by Oschlies et al. [30].

*CD25* was the least (5.3%) expressed marker as we have reported; and was co‐expressed at all times with *CD3* suggesting its presence within a T‐cell population [11, 31]. This finding is consistent with Miles et al., who reported *CD25* expression in a subset of T‐lymphoblastic lymphoma (8%) but not B‐cell lymphoma [32]. We also observed that, 50% of children who expressed *CD25* died on admission, suggesting an association with adverse clinical outcome in this cohort. However, in the absence of broader T‐cell lineage markers, no definitive conclusions can be drawn regarding specific T‐cell subsets or underlying functional immune mechanisms, which is a limitation of this study.

*BCL‐2* expression inhibits apoptosis and favors cancer progression [33] and was expressed in 55.3% of the cases. This indicates that more than half of the case we studied expressed the tumor survival‐promoting protein, potentially contributing to their resistance to therapy and more aggressive disease course which favors low survival time (22 months).

Although Burkitt lymphoma typically shows absent or low *BCL‐2* expression, a subset of our cases demonstrated positivity (36.4%). Research has shown that *BCL‐2* expression in Burkitt lymphoma is more common than traditionally recognized, challenging the diagnostic utility of this marker. Masqué‐Soler et al. found *BCL‐2* expression in 23% of 150 Burkitt lymphoma cases [34]. Similarly, Pervez et al. reported *BCL‐2* positivity in 23% of cases, including strong diffuse staining in 10.2%, although this observation was particularly in adult patients with parotid involvement [35]. These findings as well as the findings observed in this study confirm that *BCL‐2* expression occurs in a substantial subset of BL cases and reflects underlying biological heterogeneity. In a similar study, Oschlies et al. reported that, 55% of NHL cases expressed *BCL‐2* although there was no significant correlation between biomarker expression and survival [30]. In Kenya, Wawire et al. reported a much lower (40.6%) expression rate among NHL cases [36]. The higher expression rate we observed explains the aggressive nature of childhood NHL. The variations in biomarker expression could also explain the heterogeneity in NHL clinical and immunophenotypic characteristic across regions. Given the heterogeneity of BCL‐2 across NHL subtypes and the absence of additional markers such as MYC, CD10, BCL6, Ki‐67, and CD20 from the evaluated panel, BCL‐2 positivity in this cohort should be regarded as an exploratory finding.

In this study, the overall median survival time was 22 months; indicating that 50% of children diagnosed of NHL are likely to survive up to 22 months after diagnosis and initiation of chemotherapy. There was however a high degree of variability in survival times among children ranging from 3.96 months to 40.11 months. The wide degree of survival time variability could be related to differences in patient demographic and clinical characteristic of tumors which is similar to findings reported by Hesseling et al. and Choeyprasert et al. [21, 23]. Patients reported at various stages of tumor presentation at the time of diagnosis. 5 and 7 children representing 31.2% in aggregate, presented with Stages III and IV respectively which were considered advanced stages of the tumor which affect overall survival time [21, 23]. Even though Hesseling et al. [23] recorded a relatively higher proportion (71.9%) of patients presenting with advanced stages of the disease, a better (78.4 months) survival time was recorded. This was due to their ability to accurately stratify the cases using advanced immunophenotypic testing for tailored chemotherapy which was absent in the present study. It was also observed that, 22 cases were not graded. From the data available, it is our reasoning that a significant proportion of ungraded cases could be assigned advanced stages of tumor if they were graded. Considering that this assumption is true, more than half of children would present with advanced stage of the disease at the time of diagnosis and could account for the poor survival times and high mortality recorded in this study.

Additionally, as noted in the Methods section, discordant reports between pathologists required third‐party adjudication, reflecting the diagnostic complexity of NHL and the risk of initial misclassification, which may have contributed to suboptimal treatment and poorer outcomes [37].

The survival rates at specific time points provide additional perspective. At 10 months, 76% of patients survived with a relatively stronger rate (58.4%–100%). In the 2nd year at 20 months, the survival rate decreased to 61% (40.0%–92.7%), depicting a gradual decline in survival rate. In the 3rd and 4th year at months 30 and 40, the survival rates decreased significantly through 40% (16.4%–100%) to 20% (3.8%–100%) with wide variability in survival times. In Ghana, YA et al. reported a similar declining trend in survival rate over time at the Korle‐Bu Teaching Hospital even though a higher survival rate of 98.6% was recorded in the first year after diagnosis and treatment [17]. Both studies were carried out in Ghana; however, demographic and ethnic differences between the southern and middle/northern areas may have contributed to the varying reports made.

We further observed that, 11 (30.6%) died at various time intervals within the 1st year after diagnosis whiles on admission and 1 (2.8%) died shortly after discharge. We observed 10 (27.8%) patients abandoned treatment and their survival status could not be established. This finding is similar to a study carried out in Kenya where 22% of children died of Burkitt lymphoma whiles on admission and 16% of patients relapsed or died later [26]. In Nigeria, Eke et al. [22] reported a relatively higher mortality rate of 45%. Kenya, Nigeria and Ghana share similar geographic and socio‐economic characteristics with similar childhood cancer burden. The poor healthcare systems, inadequate and often under resourced laboratories exacerbate the burden of childhood cancers resulting in poor survival times.

In this study, we categorized tumors as low‐grade, high‐grade, and Burkitt lymphomas, similar to a report in Ghana by YA et al. [17]. Even though there was no significant difference between the survival rates among these categories, children with high‐grade lymphoma had a better survival rate (66%) compared to those assigned Burkitt lymphoma (38%) and low‐grade lymphoma (0%). Although further studies are required to establish the reasons for this observation, it is our reasoning that high‐grade tumors are mostly well differentiated and respond better to chemotherapy. For high‐grade lymphoma, the survival rate varied from 35.2% to 100%, indicating considerable survival uncertainty; however, a better prognosis exists compared with Burkitt lymphoma, which had a poorer survival outcome (CI, 8.4%–100%). Burkitt lymphoma is an aggressive tumor commonly associated with children. It has the propensity to spread quickly, and when not treated, it could be potentially fatal. In this study, 11 (28.9%) patients were assigned Burkitt lymphoma and were at risk of poorer survival outcome, which is similar to findings by Pedrosa et al. in Northeast Brazil [18]. Thirteen (34.2%) patients who were assigned low‐grade NHL rather had the poorest outcome, with a survival rate of 0%, indicating that none of the patients in this subgroup survived during the observed period. Although low‐grade NHL is generally associated with a more favorable prognosis, this unexpected finding may reflect the small sample size, delayed presentation, advanced disease at diagnosis, treatment‐related factors, or other unmeasured clinical characteristics.

In our study, we observed that age, gender, duration of symptoms, the expression of *CD3* and *CD25* antigenic markers, and case categorization were not significantly associated with the overall survival, which is similar to study findings in Cameroon by Hesseling et al. [23]. Contrary to our findings, Jiang et al. reported that for all patients recruited, age, male sex, and more than 3 nodal site involvement were significant factors associated with patient survival [38]. Jiang et al. [38] sample population included adults, which may have contributed to the variations observed. *BCL‐2* expression and nodal/extra‐nodal involvement were, however, significant factors associated with NHL survival in children. High level expression of *BCL‐2* by lymphoma cells was linked to poor prognosis and inferior survival (*p* = 0.030). This finding is similar to study observations reported by Siddiqui et al. and Wawire et al. [34, 36]. Contrary to these findings, Oschlies et al. reported no significant correlation between *BCL‐2* expression and survival outcome in Germany [30]. Oschlies et al. [30] could not assign clear reasons to the observations; however, *BCL‐2* expression produces proteins that downregulate apoptosis and promote chemoresistance, which may partly explain the poor prognosis and inferior survival observed in the current study. Our findings have also demonstrated that the site of tumor (nodal or extra‐nodal involvement) significantly correlates (*p* < 0.001) with NHL survival. This finding is similar to a study by Jiang et al. who reported that multiple nodal involvement and spread of tumor cells was an independent predictive factor associated with survival [38]. NHL spread to other organs suggests more aggressive and advanced disease. The Dukes and Murphy classification systems for pediatric NHL employ the extent of nodal and/or extra‐nodal involvement in staging the tumor. Advanced tumor stages (Stages III and IV) characterized by multiple extra‐nodal involvement correlate well with poor survival. This could explain the finding of the current study.

Although this study offers valuable insights into biomarker expression and survival rates among children with NHL, it has limitations. A significant proportion of tissue blocks could not be retrieved from the pathology archives due to poor archiving systems, which reduced the sample size. This consequently limited the statistical power of the study, potentially affecting the detection of significant associations and the generalizability of the findings. The retrospective design limited the ability to control and standardize chemotherapy and other clinical care practices, which are critical factors in overall survival. Patients who survived and were discharged were not followed up to assess their long‐term survival status. The relatively high rate of treatment abandonment (27.8%) and incomplete follow‐up may have limited complete ascertainment and, consequently, affected the accuracy of the survival estimates. Thus, making it underpowered for multivariable Cox proportional hazards modeling. Therefore, the survival findings should be interpreted with caution. Moreover, this study categorized NHL cases based on clinical aggressiveness rather than comprehensive WHO/ICC diagnostic classification. As a result, biological heterogeneity within the low‐grade and high‐grade groups may have been overlooked, and the findings should be interpreted as comparisons between broad clinical categories rather than specific lymphoma subtypes. Finally, the immunophenotypic panel used in this study did not include B‐cell lineage markers such as *CD20, MYC, CD10, BCL6*, and *Ki‐67*, limiting comprehensive immunophenotypic characterization and precise lineage assignment of NHL cases. In addition, since most cases were of B‐cell origin, the prognostic role of *CD3*, *CD25*, and BCL2 in T‐cell NHLs could not be adequately evaluated. Future studies with larger cohorts and more balanced subtype representation are warranted to clarify the prognostic implications of these markers in T‐cell NHLs.

## 5 Conclusion

Overall survival among NHL patients in Ghana was poor, with a median overall survival of 22 months. Most patients presented with advanced disease, including nodal and extranodal involvement, and had limited access to radiotherapy and palliative care. *BCL‐2* was the most frequently expressed biomarker, and its expression, together with the extent of tumor spread, showed significant associations with survival. Identifying prognostic factors that facilitate early diagnosis and treatment is essential for improving survival outcomes among NHL patients in this setting.

## Author Contributions

**Samuel Asamoah Sakyi:** conceptualization, methodology, project administration, supervision, formal analysis, writing – review and editing, validation. **Bernard Gyan:** methodology, writing – original draft, investigation. **Ernest Adankwah:** conceptualization, writing – review and editing, validation, supervision. **Ernest Kwasi Adjei:** validation, writing – review and editing, methodology. **Vivian Paintsil:** validation, writing – review and editing, data curation, investigation. **David Kwabena Adu:** validation, writing – review and editing, data curation. **Manu Akwasi Owusu:** validation, writing – review and editing, data curation. **Clement Binwatin Dagungong:** validation, writing – review and editing, data curation. **Muniru Mohammed Tanko:** validation, writing – review and editing, data curation. **Kervin Edinam Zogli:** validation, writing – review and editing. **Alfred Effah:** data curation, formal analysis, validation, visualization, writing – review and editing, methodology, writing – original draft. **Samuel Kwame Sopuruchi Agomuo:** validation, methodology, writing – review and editing, visualization, formal analysis. **Samuel Kwarteng:** validation, visualization, formal analysis, methodology, writing – review and editing. **Beatrice Nyantakyiwaa:** writing – review and editing, methodology, validation. **Edward Osei Gyimah:** writing – review and editing, visualization, methodology, data curation, writing – original draft. **Harriet Yeboaa Diawuo:** writing – review and editing, data curation, investigation, methodology. **George Oduro Owusu:** writing – review and editing, formal analysis, methodology. **Ebenezer Senu:** writing – review and editing, formal analysis, data curation, visualization, validation. **Oscar Simon Olympio Mensah:** writing – review and editing, data curation, formal analysis, validation. **Stephen Opoku:** formal analysis, writing – review and editing, data curation, visualization, validation. **Benjamin Amoani:** writing – review and editing, formal analysis, conceptualization, validation, supervision. **Linda Ahenkorah Fondjo:** conceptualization, formal analysis, writing – review and editing, validation, supervision.

## Funding

The authors have nothing to report.

## Ethics Statement

Approval to conduct the study was sought from the Committee on Human Research Ethics and Publication, KNUST (CHRPE/AP/767/22) and the Institutional Review Board (IRB) of KATH (KATH IRB/AP/122/22). Patient confidentiality was maintained throughout by anonymizing all data extracted from the records. This study was carried out in accordance with the Declaration of Helsinki (2000) of the World Medical Association.

## Consent

The authors have nothing to report.

## Conflicts of Interest

The authors declare no conflicts of interest.

## Data Availability

All data generated or analyzed during this study are included in this article and can be requested from the corresponding author.
